# Draft Genome Sequence of *Elizabethkingia meningoseptica* Isolated from a Traumatic Wound

**DOI:** 10.1128/genomeA.00355-14

**Published:** 2014-05-08

**Authors:** Joshua Quick, Chrystala Constantinidou, Mark J. Pallen, Beryl Oppenheim, Nicholas J. Loman

**Affiliations:** a Institute of Microbiology and Infection, University of Birmingham, Birmingham, United Kingdom; b NIHR Centre for Surgical Reconstruction and Microbiology, Queen Elizabeth Hospital, Birmingham, United Kingdom; c Division of Microbiology and Infection, University of Warwick, Coventry, United Kingdom

## Abstract

We report the draft genome assembly of *Elizabethkingia meningoseptica* strain 502. The sample was isolated from the wound of a repatriated military serviceperson who suffered major trauma from an improvised explosive device (IED), resulting in wounds with extensive environmental contamination. *E. meningoseptica* was isolated from wounds in both legs. The draft genome assembly has 21 contigs with a total size of 3,960,744 bases. The genome contains genes encoding 26 putative β-lactamases.

## GENOME ANNOUNCEMENT

*Elizabethkingia meningoseptica* (previously *Chryseobacterium meningosepticum, Flavobacterium meningosepticum*) is a ubiquitous Gram-negative bacterium, which is associated with meningitis in premature neonates and immunocompromised patients (1–3). Here, we report the draft whole-genome sequence of *E. meningoseptica* 502. This strain was isolated from the wounds of a military patient who suffered major trauma from an improvised explosive device (IED) in Afghanistan. As part of the standard military care pathway, the patient was repatriated to the Queen Elizabeth Hospital in Birmingham, United Kingdom, where *E. meningoseptica* was detected through routine clinical microbiological diagnosis.

DNA for sequencing was extracted and purified using a DNeasy extraction kit (Qiagen, Venlo, Netherlands) from a pure colony isolate of *E. meningoseptica*. The Illumina Nextera sample preparation kit (Illumina, Great Chesterford, United Kingdom) was used to prepare a sequence-ready library. Sequencing was performed on an Illumina MiSeq instrument with a version 1 300-cycle kit. The reads were adaptor- and quality-trimmed using Trimmomatic-0.30 (4). Trimming occurred if the Nextera adapter sequence was detected at the 3′ end of the read or if the base quality dropped below a Phred score of 20 (99% accuracy). A *de novo* assembly was generated using SPAdes-2.5 (5) with *k*-mer values 55, 77, 99, and 127. The reads were then remapped to the assembly with BWA version 0.7.5a-r405 to calculate the read depth. The draft genome was annotated automatically using the NCBI Prokaryotic Genome Automatic Annotation Pipeline (PGAAP) (6). Putative β-lactamases predicted by PGAAP were subsequently searched by using BLASTp (7) against the MBLED database (8) and by using online domain searches against the Pfam database of protein families (http://pfam.sanger.ac.uk).

Sequencing generated 1,014,092 paired-end reads, with a mean insert size of 404 bases. After adapter and quality trimming, the reads had a mean length of 146 bases. The resulting assembly consisted of 21 contigs longer than 200 bases, with an *N*_50_ size of 482,386 bases, a total size of 3,960,744 bases, and an average G+C content of 35.85%. The mean coverage depth was 71×. Remapping the reads to the annotated ribosomal 16S sequence gave a coverage depth of 357×, suggesting that the genome contains five copies of the rRNA operon. No identical match for the 16S rRNA gene was found; however, a sequence differing by a single base has been submitted for *E. meningoseptica* isolated from a mudfish (accession no. AY683476).

Multiple antibiotic resistance-associated coding sequences were detected, including sequences encoding 26 putative β-lactamases. This supports the previous suggestion that *Elizabethkingia* species might act as an environmental reservoir of novel β-lactamases (9). These coding sequences were homologous to five distinct families of β-lactamase genes defined by Pfam (http://dx.doi.org/10.6084/m9.figshare.980684). The presence of these genes is consistent with the laboratory antibiogram results, showing the strain to be highly resistant to meropenem, with an MIC of >32 mg/liter.

### Nucleotide sequence accession numbers.

This whole-genome shotgun project has been deposited in GenBank under the accession no. AVCQ00000000. Short reads have been deposited in the ENA under the accession no. PRJEB6023.
